# Physicochemical and Biological Properties of Graphene-Oxide-Coated Metallic Materials

**DOI:** 10.3390/ma14195752

**Published:** 2021-10-01

**Authors:** Aleksandra Poniatowska, Paulina Anna Trzaskowska, Maciej Trzaskowski, Tomasz Ciach

**Affiliations:** 1 Faculty of Chemical and Process Engineering, Warsaw University of Technology, Warynskiego 1, 00-645 Warsaw, Poland; tomasz.ciach@pw.edu.pl; 2 Centre for Advanced Materials and Technologies CEZAMAT, Warsaw University of Technology, Poleczki 19, 02-822 Warsaw, Poland; paulina.trzaskowska@pw.edu.pl (P.A.T.); maciej.trzaskowski@pw.edu.pl (M.T.)

**Keywords:** biocompatible coatings, metal implants, electrophoretic deposition, graphene oxide, stainless steel 316L, chemical modification of gold

## Abstract

In this article, we present graphene oxide (produced by a modified Hummers’ method) coatings obtained using two different methods: electrophoretic deposition on 316L stainless steel and chemical modification of the surface of gold applied to the steel. The coating properties were characterized by microscopic and spectrometric techniques. The contact angle was also determined, ranging from 50° to 70°. Our results indicated that GO coatings on steel and gold were not toxic towards L929 cells in a direct cell adhesion test—on all tested materials, it was possible to observe the growth of L929 cells during 48 h of culture. The lack of toxic effect on cells was also confirmed in two viability tests, XTT and MTT. For most of the tested materials, the cell viability was above 70%. They showed that the stability of the coating is the crucial factor for such GO coatings, and prove that GO in the form of coating is non-toxic; however, it can show toxicity if detached from the surface. The obtained materials also did not show any hemolytic properties, as the percentage of hemolysis was on the level of the negative control, which is very promising in the light of future potential applications.

## 1. Introduction

Metals have been used in implantology for over a hundred years; the usage of steel to join bone fractures together was first introduced into the human body in 1895. Since then, the field of biomedical applications of metals has been constantly developing [1,2]. An example of the use of metals may be stainless steel used in cardiovascular diseases, or titanium and cobalt alloys used in orthopedics. Metallic biomaterials owe their popularity to excellent mechanical properties, but they can corrode and are not biologically active [3]. For this reason, scientists are constantly working on solutions that would minimize emerging problems.

Many research groups are working on graphene oxide coatings on metals that could be used in various fields, especially in directions such as fuel cells [4] or extraction [5]. It has also gained interest in surface engineering as a potential compound of implants, as carbon-based coatings have been successfully applied in, e.g., cardiovascular devices [6,7]. It has been reported that coatings on stainless steel which are comprised of graphene oxide have been shown to improve anti-corrosion properties [8,9] and biofouling resistance [10,11]. There are also studies on GO coatings on titanium that indicate their improvement of the physicochemical properties of the obtained materials [12,13]. GO can also be used to modify other surfaces, e.g., polymer surfaces, in the production of scaffolds [14,15]. Other studies show that graphene oxide as an intermediate layer in coatings, in combination with heparin and poly(3,4-ethylenedioxythiophene) [16] or drugs, i.e., docetaxel [17], may improve the biocompatibility of steel stents and reduce platelet adhesion. Still, the status of graphene oxide as a biomaterial remains unsettled. It cannot be claimed that it generally enhances the biocompatible properties of biomedical surfaces. Researchers agree that its effect on living cells depends on its properties, such as the numbers of layers, size, form, and the content of chemical groups that emerge from diverse synthesis parameters applied [18,19].

In the present study, we examined the application of graphene oxide, synthesized by our group with a modified Hummers’ method, as a biocompatible coating on metals. We focused on finding simple and effective methods to achieve such coatings on stainless steel and gold. Two different techniques of coating were applied: electrodeposition and covalent bonding. Obtained coatings were examined in terms of their physicochemical and biological properties. Finally, we studied the biocompatibility of such obtained materials.

## 2. Materials and Methods

### 2.1. Materials

Sodium hydroxide, 98% phosphoric acid, 96% sulfuric acid, 30% hydrogen peroxide, 37% hydrochloric acid, 96% ethanol, and potassium permanganate were purchased from Chempur (Piekary Śląskie, Poland) and used as received. 1-Ethyl-3-(3-dimethylaminopropyl)carbodiimide (EDC), sulfo-N-hydroxysuccinimide (sulfo-NHS), cystamine, and Triton X-100 were purchased from Sigma-Aldrich (Saint Louis, MO, USA). For biological studies, we used DMEM medium with\without Phenol Red (Gibco, Waltham, MA, USA), fetal bovine serum (Gibco), 1% penicillin-streptomycin solution (Gibco), trypsin/EDTA (0.25%, Gibco), XTT cell proliferation kit ((2,3-Bis-(2-Methoxy-4-Nitro-5-Sulfophenyl)-2H-Tetrazolium-5-Carboxanilide, Roche), MTT cell proliferation assay (Thiazolyl Blue Tetrazolium Bromide, Sigma-Aldrich), Alexa Fluor 488 Phalloidin (ThermoFisher, Waltham, MA, United States), DAPI (ThermoFisher), human serum albumin (sera and vaccines manufacturing company BIOMED, Warsaw, Poland), and phosphate-buffered saline (Gibco).

### 2.2. Synthesis and Characterization of Graphene Oxide

Graphene oxide (GO) was synthesized by the method presented by Marcano et al. [20]. First, 1 g graphite and 6 g KMnO_4_ were added to a vessel, followed by 13 mL H_3_PO_4_ and 120 mL H_2_SO_4_. The vessel was placed in a water bath and stirred until a temperature of 25 °C was reached. After this stage, the suspension was heated to 50 °C and left for 24 h under continuous stirring. After this time, the reaction vessel was cooled down to room temperature and about 300 g of ice was added to it, which caused a heating up of the mixture. After cooling to room temperature again, residual permanganate was removed by adding 30 mL of 30% H_2_O_2_ to the suspension. The suspension was centrifuged (4000 rpm for 30 min) and then the precipitate was suspended in water. The washing and centrifugation processes were repeated 5 times (2×water, 30% HCl, and 2× 96% ethanol). The final product was an aqueous GO suspension of GO flakes with a size of about 2–5 µm, whose other properties we have reported previously [21]. Before the coating process, the obtained powder of graphene oxide was characterized by Raman spectroscopy (Thermo Scientific Nicolet Almega XR Dispersive Raman Spectrometer).

### 2.3. Preparation of Stainless Steel and Gold Substrates

For studies, discs made from stainless steel 316L of a diameter of 14 mm, prepared by STOMILEX, Piastów, Poland, were used. Then, the steel discs were polished using the rotary method by MARBAD, Warsaw, Poland. The discs were placed in a rotating tumbler together with ceramic fittings of a diameter of 6 mm, together with a non-ionic detergent for 18 h. Before being subjected to the coating process, the discs were washed in a 3:1 solution of water and acetone using ultrasound (Hielscher, Teltow, Germany). The washed discs were dried at 40 °C in a laboratory dryer. As a substrate for the preparation of GO coatings on gold, polished and washed steel discs were sputter-coated with a 25 nm layer of pure gold with the use of a Quorum plasma coater.

### 2.4. Electrophoretic Deposition of Graphene Oxide on a Stainless Steel Surface

**Figure 1:**
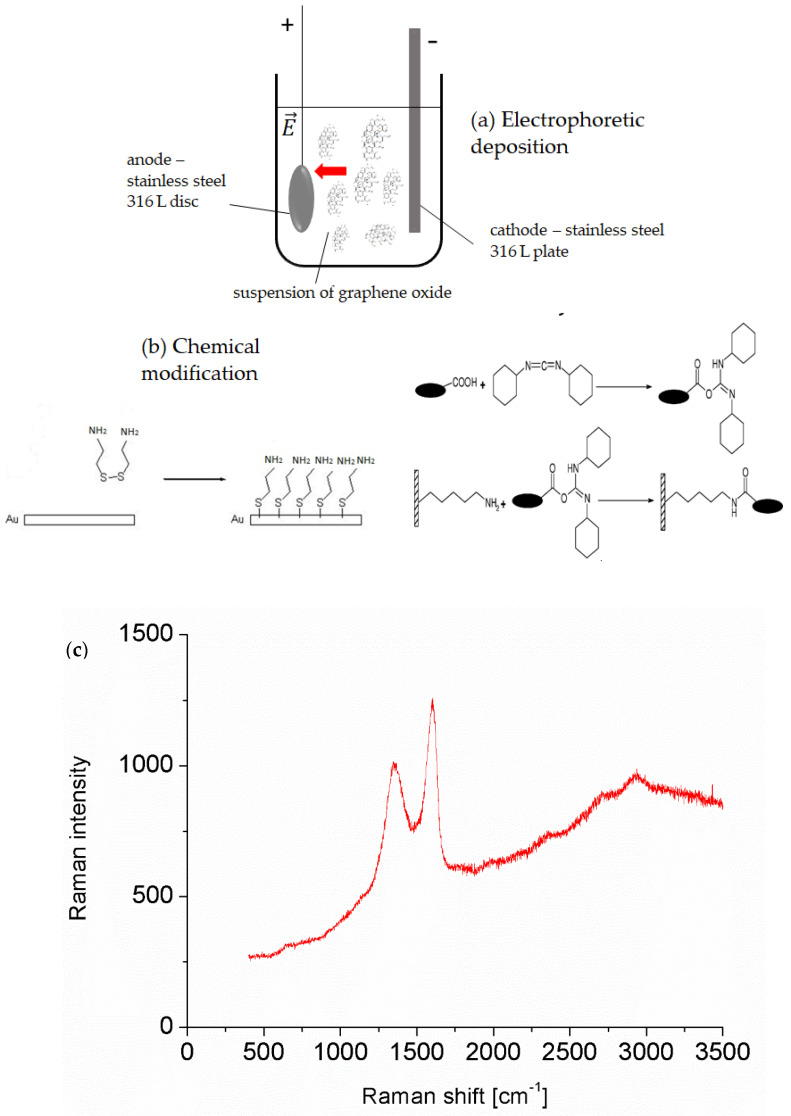
(**a**) Scheme of electrophoretic deposition set, (**b**) scheme of the coating process of gold via the chemical method, and (**c**) Raman spectrum of obtained graphene oxide.

For electrophoretic deposition, a laboratory power supply from Keysight Technologies (Santa Rosa, CA, USA)) E349A was used. GO suspensions of 0.1% w/w in water (variant A) or a mixture of water and ethanol in a ratio of 1:1 (B) and 3:1 (C) were prepared in 100 mL plastic vessels. All suspensions were sonicated for 5 min just before the coating process (Hielscher). Then, two electrodes were placed in the vessel: a steel disc to be coated (anode) and a counter electrode (steel plate, cathode) (Figure 1a). The container was placed on a magnetic stirrer (stirring speed 500 rpm) for 30 min by 2 mA and 3–6 V.

### 2.5. Chemical Modification of the Gold Surface with Graphene Oxide

A two-stage process of GO bonding to the gold surface using a cystamine linker was developed. In the first stage, the gold surface was coated with a monolayer of the linker. It was produced by the dip-coating method through the immersion of the substrate to be coated in an aqueous solution containing the linker—cystamine (0.1%). The covalent binding of cystamine to the gold surface was achieved via thiol groups present in cystamine. In the second stage, amide bonds between cystamine molecules present in the monolayer and carboxylic groups of GO were obtained (Figure 1b). To carry out this reaction, to a water suspension of GO, EDC and sulfo-NHS were added, resulting in final concentrations of 0.5% *w*/*w* GO, 2 mM EDC, and 5 mM sulfo-NHS. In the reaction, carboxylic groups of graphene oxide were activated using carbodiimide and then stabilized as an ester with the use of sulfo-N-hydroxysuccinimide, which then easily reacts with amine groups present in the system only on the cystamine monolayer surface. As a comparison, similar gold-surface discs coated with GO by means of a simple dip-coating method in the 0.5% *w*/*w* suspension of GO without any linker were also investigated.

### 2.6. Physicochemical Properties of GO-Coated Metals

The obtained GO coatings were characterized in terms of chemical composition by using a Nicolet FTIR-ATR Spectrometer (Thermo Scientific) with OMNIC 8.0 software, and imaged in high magnification with the use of a Hitachi SU 8230 electron microscope (Hitachi High Tech.,Tokyo, Japan). The contact angle was measured by the use of the sessile drop method (KRUSS goniometer DSA100S, Hmaburg, Germany). Briefly, on each material, 5 μL droplets of distilled water were placed (3 droplets per one disc). Preliminary GO coating durability tests were also carried out. The coated steel discs with PBS in a 24-well plate were placed at 37 °C on a microplate shaker (200 rpm) for 1, 2, and 7 days. Each disc was placed in 0.7 mL of PBS. After this time, the coatings were characterized by infrared spectroscopy.

### 2.7. Biological Properties of GO-Coated Metals

Before each of the following tests, the obtained coated disks were sterilized by being dipped in 70% ethanol and washed with sterile phosphate-buffered saline (PBS) at room temperature for 24 h. For the investigation of biological properties (cell viability tests, cell adhesion), mouse fibroblast cell line L929 (Sigma Aldrich) was used. The cells were precultured with DMEM medium containing Phenol Red (Gibco) and 4 mM of L-Glutamine, supplemented with 10% serum (fetal bovine serum, Gibco) and 1% penicillin-streptomycin solution (Gibco). Before viability tests, cells were subcultured using trypsin/EDTA (0.25%, Gibco) and seeded in a 96-well plate with a final concentration of 10^5^ cells/mL using Phenol-Red-free DMEM (Gibco), supplemented as described. The cells were incubated at 37 °C, 5% CO_2_ for 24 h to achieve subconfluency.

#### 2.7.1. Cell Viability Tests

Two viability tests with the use of the XTT cell proliferation kit ((2,3-Bis-(2-Methoxy-4-Nitro-5-Sulfophenyl)-2H-Tetrazolium-5-Carboxanilide, Roche) and MTT cell proliferation assay (Thiazolyl Blue Tetrazolium Bromide, Sigma Aldrich) were conducted. According to ISO 10993-5 standard [22], the extracts were prepared in duplicates of each variant of material by incubation in a supplemented DMEM medium w/o Phenol Red (1 mL per 3 cm^2^ of disc surface, according to ISO 10993-12) [23] for 24 h (37 °C, 5% CO_2_). After that time, extracts were added to cells and they were incubated for the next 24 h. As a negative and positive control (NC and PC, respectively), DMEM medium w/o Phenol Red (NC) and DMEM medium w/o Phenol Red with 0.1% (v/v) Triton X (Sigma Aldrich) (PC) were used. In the next step, extracts were removed and XTT or MTT solution (1 mg/mL in DMEM without supplements and Phenol Red) was added. After 4 h of incubation, in the case of the XTT test, the absorbance of resulting solutions was measured with the use of a UV–Vis plate reader (Epoch, BioTek, Winooski, VT, USA, Gen5 software) at 470 nm with a reference wavelength at 650 nm. In the case of the MTT test, the solutions were removed from wells and the crystals were dissolved in 100 µL of isopropanol. The absorbance was measured at 570 nm, reference wavelength 650 nm.

#### 2.7.2. Protein Adhesion on Surfaces of Obtained Materials

To determine the interaction of the prepared materials with protein occurring in blood plasma, a test was performed according to a previously described procedure [24]. The obtained materials (2 for each variant) were incubated in 500 µL of saline (PBS, pH = 7.4) at 37 °C for 1 h and then for another 1 h in 500 µL of 4 mg/mL human serum albumin (sera and vaccines manufacturing company, BIOMED) solutions in PBS. Afterwards, a BCA protein assay kit (Sigma Aldrich) was used to determine the number of absorbed proteins.

#### 2.7.3. Hemolysis

According to the protocol suggested by the ASTM F756-13 standard [25], the GO-coated discs were incubated with 1 mL of 0.9% NaCl each at 37 °C for 1 h in a 24-well plate. Next, the materials were put into a new 24-well plate and 1 mL of fresh NaCl per well was added, followed by the addition of 0.14 mL of human blood, which was freshly collected in K_2_ EDTA tubes and dissolved 10× to the desired hemoglobin content. The negative (NC) and positive (PC) controls were 0.14 mL of blood mixed with 1 mL of 0.9% NaCl (NC) and 0.14 mL of blood mixed with 1 mL of distilled water (PC), respectively. Samples were gently shaken and incubated at 37 °C for 3 h. After that, the solution from each well was transferred to a separate Falcon tube and centrifuged at 2500 rpm for 5 min. In the end, the absorbance of the samples was measured at 545 nm. The hemolysis ratio was calculated according to the following equation:

$$\%H=\frac{Abs_{sample}-Abs_{NC}}{Abs_{PC}-Abs_{NC}}\cdot 100\%$$

#### 2.7.4. Cell Adhesion on Surfaces of Obtained Materials

For the cell adhesion test, the GO-coated discs were incubated together with L929 cells in 24-well plates. The materials were put into wells and then the cells were suspended in a medium (Phenol-Red-free DMEM (Gibco) supplemented as described above), with the final concentration of 10^5^ cells/mL seeded on the materials. Plates were placed in an incubator for 24 h and 48 h. After this time the adhered cells on the surface of the materials were fixed with paraformaldehyde (4% in PBS), washed 4 × 5 min in PBS, and permeabilized in 0.2% Triton X-100 in PBS for 8 min. In the next step, the cells on materials were dyed with the use of Alexa Fluor 488 Phalloidin (AF488) (ThermoFisher, A12379) and DAPI (ThermoFisher, D3571), according to the manufacturer’s instructions. The materials were incubated in the dark with 200 µL 0.025% AF488 in PBS per well, washed 4 × 5 min in PBS, and incubated in 200 µL of 300 nM DAPI in PBS per well, and at the end were washed 4 × 5 min in PBS. Washing was carried out on a microplate shaker at 800 rpm. Stained cells on the materials were characterized using a confocal microscope (Zeiss LSM 880, Oberkochen, Germany).

## 3. Results

Henceforth, the following symbols represent variants of material used in the study (Table 1).

### 3.1. Chemical Characterization of Graphene Oxide and Obtained Coatings

After the synthesis of graphene oxide, the dried powder was subjected to Raman spectroscopy. The obtained spectrum (Figure 1c) shows the most characteristic bands at 2938 cm^−1^—band S3, around 2700 cm^−1^—band 2D, 1604 cm^−1^—band G, and 1351 cm^−1^—band D, which is identical to the data in the literature [26]. The high G band is a result of the vibration of sp2 carbon atoms, while band D is responsible for defects in the hexagonal carbon skeleton, including the presence of oxygen groups [27].

**Figure 2:**
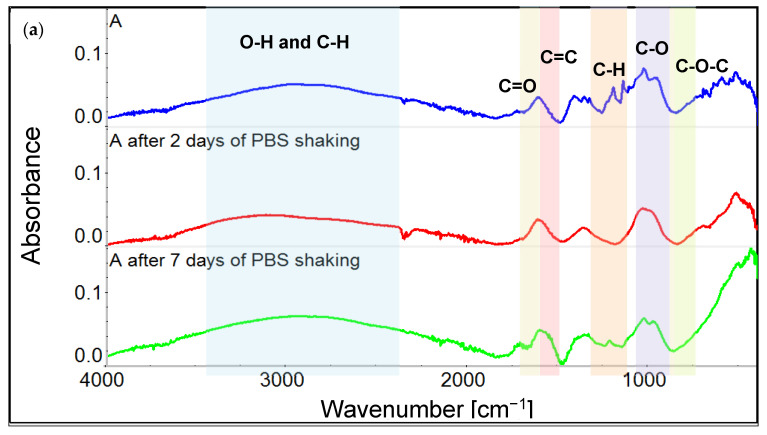

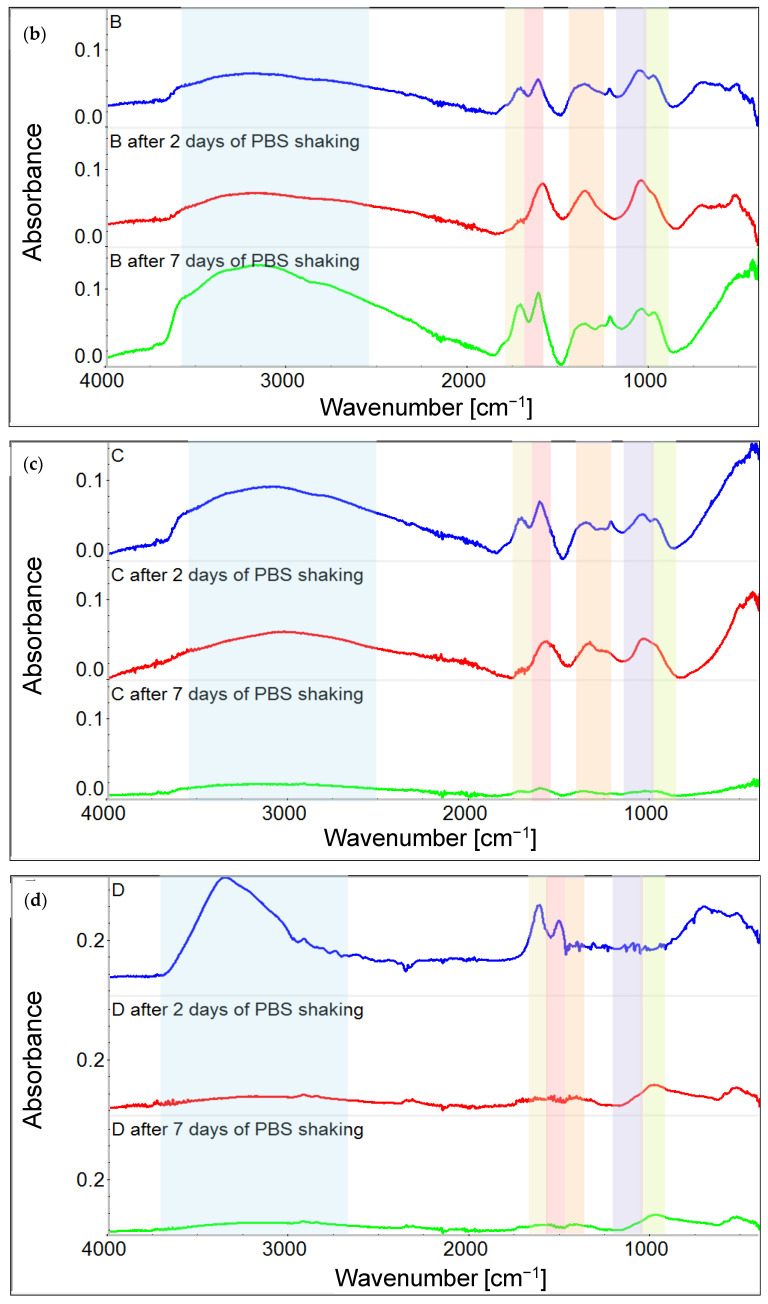

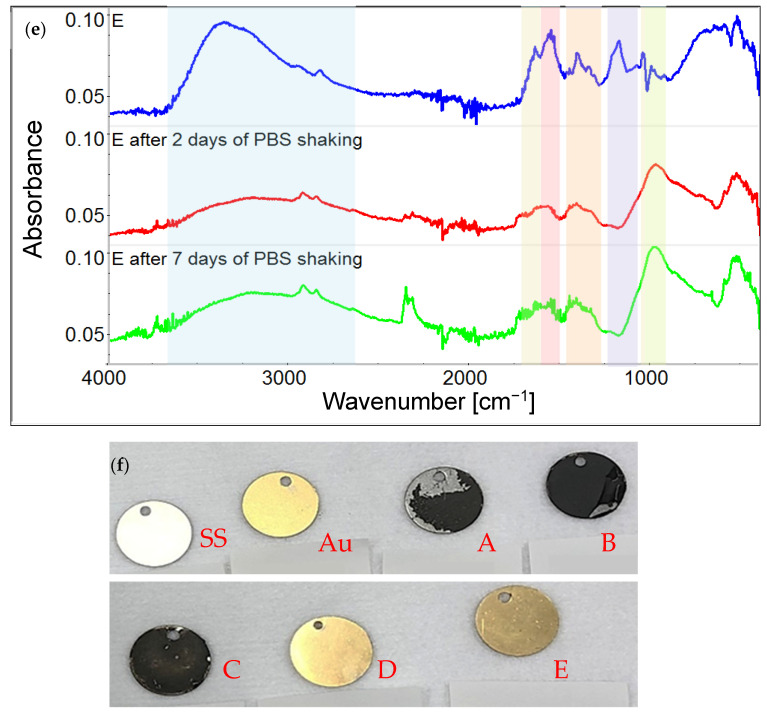
FITR spectra for metals coated with GO immediately after preparation, after 2 days, and after 7 days of PBS shaking. (**a**) Variant A, (**b**) variant B, (**c**) variant C, (**d**) variant D, and (**e**) variant E. (**f**) Pictures of all sample variants after 48 h of washing. Visible losses in coatings after washing, especially in variant A.

FTIR spectra of GO coatings on stainless steel obtained with an electrochemical method showed peaks at 2900 cm^−1^, 1700 cm^−1^, 1650 cm^−1^, 1400 cm^−1^, 1150 cm^−1^, and 1012 cm^−1^, which were associated with C-H, C=O, C=C, C-H, C-O, and C-O-C bonds, respectively. The broad-stretching vibration band around 3500 cm^−1^ came from hydroxyl groups in GO, Figure 2 (Figure 2f shows a picture of the tested materials after washing).

FITR analysis results show (Figure 2a–c), that after 48 h of incubation with PBS, there were no clear changes in the spectra of GO coatings, and that all characteristic signals appear. After 7 days, changes in peak intensity for all variants are observed, as well as partial disappearance of the hydroxyl group peak; however, signals for C=O, C=C, C-O-C can still be recognized.

Similar results have been obtained for gold-surface materials coated with GO (Figure 2d,e). Main peaks, coming from OH and C=O groups, were clearly visible for coated samples. The FTIR spectra of coated gold samples after 2 days and 7 days of washing are presented below (Figure 2d,e). In most cases the washing caused a decrease in the peak intensity; however, it did not change much between short- and long-term washing.

### 3.2. SEM Imaging

**Figure 3:**
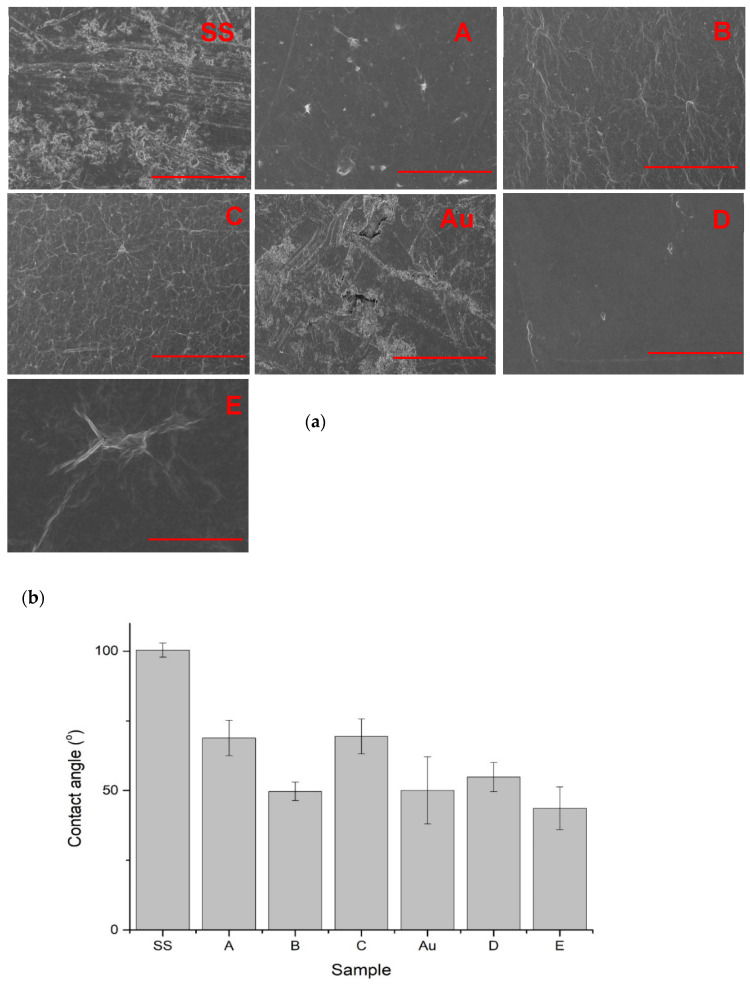
(**a**) SEM images of surfaces of obtained materials (variant numbers are shown in red in the upper-right corner), reference bar for SS, A, B, C, and D is 50 µm and for E is 5 µm. (**b**) Graph showing the decrease in contact angle with water for the treated sample.

SEM images (Figure 3) presented significant differences between the uncoated steel surface and steel with a GO coating applied. Uncoated steel showed numerous scratches that were not observed for coated surfaces (Figure 3a). On the coated surfaces, numerous “wrinkles” were visible, which may be the result of uneven application of GO flakes during electrophoretic deposition as well as the drying process. The least uneven surface was observed for coatings obtained during the deposition of GO from an aqueous suspension (variant A).

### 3.3. Contact Angle Measurements

Contact angle measurements (Figure 3b) showed that the presence of graphene oxide coatings on the steel surface significantly improved its wettability and reduced the contact angle by approximately 30% (50% for variant B). This phenomenon can be explained by the presence of a large number of hydrophilic hydroxyl and carboxyl groups incorporated in graphene oxide, which was also confirmed by FTIR. Additionally, GO filled in the scratches on the SS surface that decreased the surface energy. Contact angle values of gold samples were not significantly changed after coating with GO, since gold sputtering of SS already caused a strong contact angle decrease. It can be explained by smoothening the SS surface in the gold-sputtering process. However, the contact angle of variant E (GO covalently grafted to Au surface) was noticeably reduced.

### 3.4. Cell Viability

**Figure 4:**
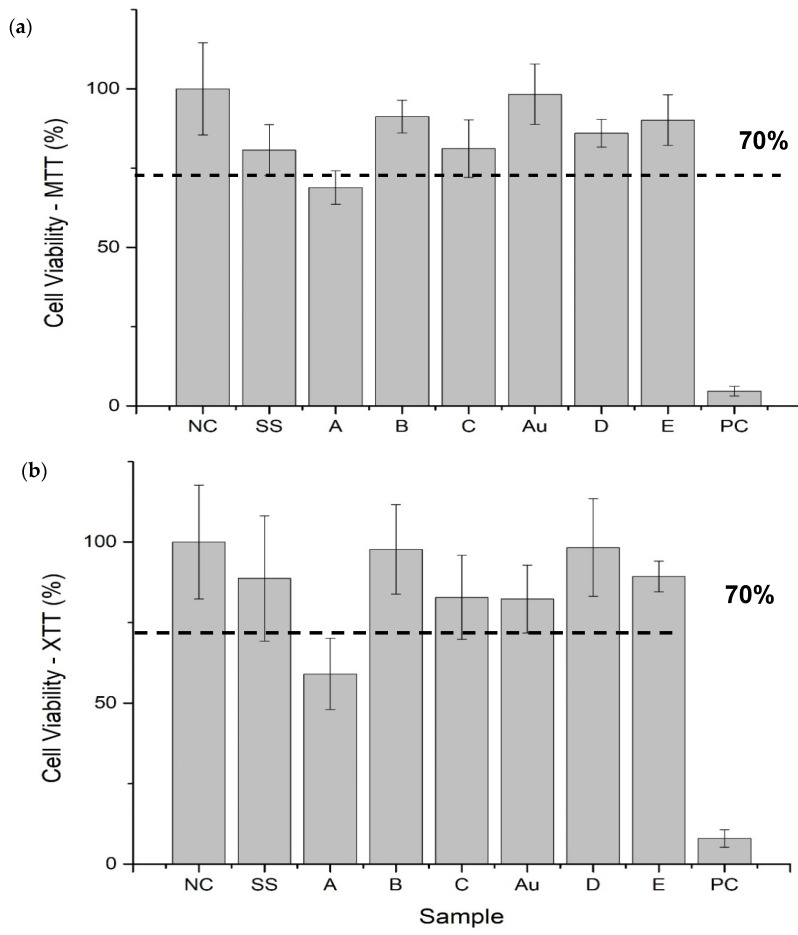
Results of the (**a**) MTT and (**b**) XTT tests for obtained materials.

Results of cytotoxicity tests performed with the use of MTT and XTT (Figure 4a,b) methods are shown below. The tests have been performed in the presence of a negative control (NC) and positive control (PC) as shown on the graphs. The results indicated that most of the materials did not exhibit cytotoxic properties. The exception, sample A (steel coated with GO through electrodeposition using the aqueous solution), was denoted to be cytotoxic in both viability assays. The samples comprised of GO electrodeposited on steel from the ethanol–water solution—B and C samples—showed the same or higher viability results than bare steel (SS). Sample B seemed to prevent the cytotoxic effect the most. GO-coated gold samples (D and E) exhibited a similar non-toxic effect on cells as clean Au.

### 3.5. Protein Adhesion on Surfaces of Obtained Materials

**Figure 5:**
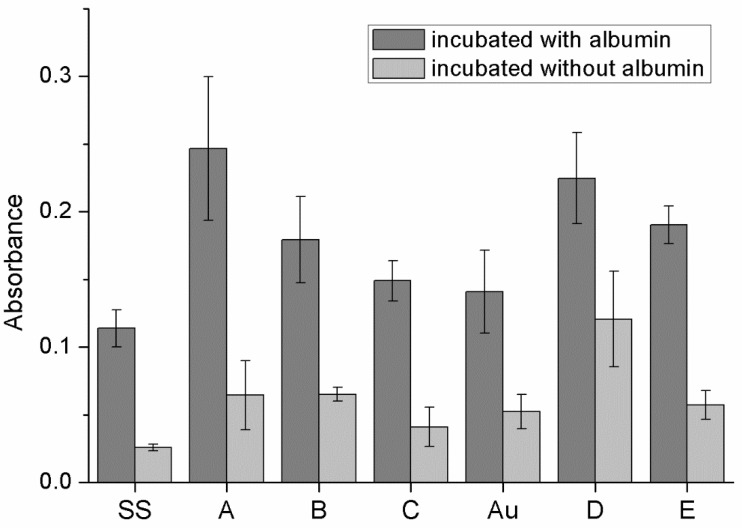
Protein adhesion test results.

The results of albumin adhesion tests are presented below. (Figure 5) The BCA assay indicated that a lower amount of albumin was adsorbed by bare metals—steel and gold—than on metallic surfaces coated with GO, regardless of the method of coating. BCA was also performed on all variants that were not incubated with albumin. The goal of such a procedure was to evaluate whether GO itself gives false-positive BCA results. It was established that the GO indeed reacted with BCA assay compounds; nevertheless, that did not affect the results trend.

### 3.6. Hemolysis

The results of the hemolysis tests of the examined materials are presented in the table below (Table 2). According to these results, none of the examined materials caused hemolysis of blood. The absorbance results obtained in the hemolysis test (Table 2) were essentially the same as for the negative control for all tested materials. Therefore, it can be assumed that the obtained hemolysis percentages were equal or close to zero.

### 3.7. Cell Adhesion

**Figure 6:**
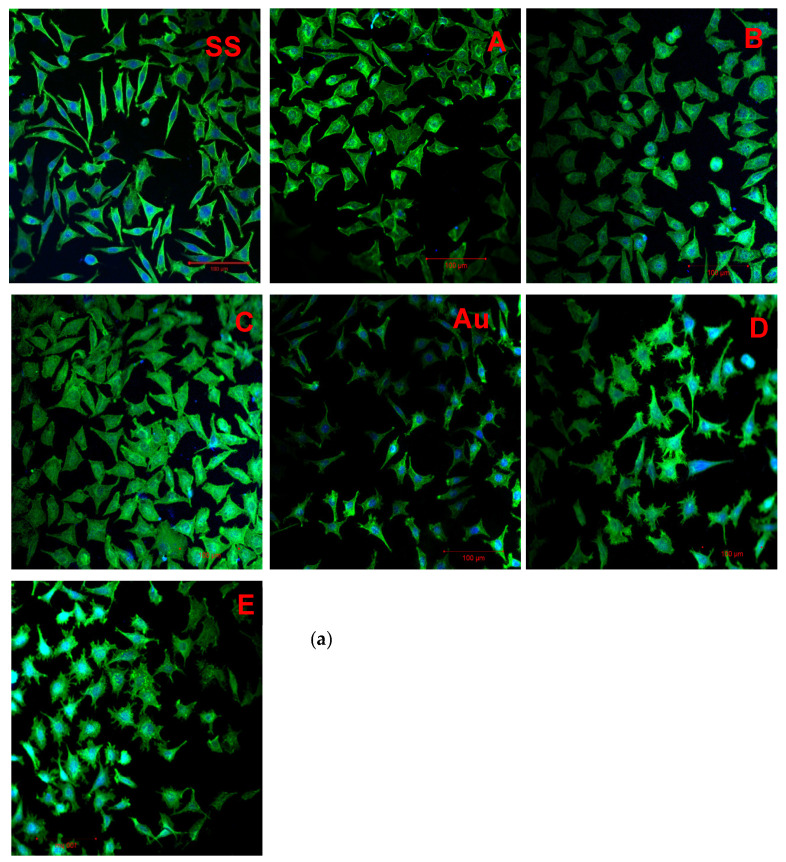

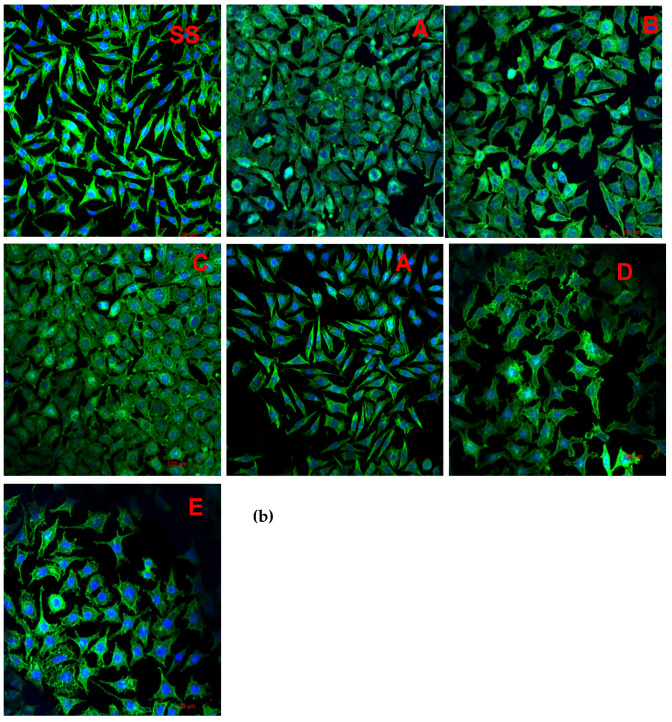
Cell adhesion on obtained materials after (**a**) 24 h and (**b**) 48 h of incubation with cells (variants are shown in red in the upper-right corner).

Images of cells adhered onto the surfaces of the examined materials are shown below. Incubation times of 24 h and 48 h have been applied. The images (Figure 6a,b) show that all the examined materials’ surfaces were appropriate for cell growth and proliferation. Additionally, differences in cells morphology on SS and other sample variants can be noticed. Cells spreading on SS were elongated rather than flattened. As can be seen in Figure 6b, after 48 h of culture, cells proliferated and covered most of the space on the surface of samples A and C. As for Au and GO-coated Au, it can be seen that L929 cells also grew and proliferated on those surfaces. The cell morphology on gold samples grafted with GO (D and E) showed differently distributed focal adhesion points than in cells growing on the surface of bare Au. Overall, more cells were present on GO–steel materials (A, B, and C) than on GO–gold materials (D and E).

## 4. Discussion

### 4.1. Chemistry of GO and Physicochemical Properties of GO-Coated Metals

Raman spectroscopy of GO applied in this research indicated the presence of O atoms and sp2-hybridized C atoms, which proved the conversion of graphite into GO. Compared to the Raman studies by Z. Liu et al., which characterized GOs with different oxygen contents, the obtained intensity ratios I_D_/I_G_ = 0.74 and I_S3_/2_D_ = 1.15 (usually used to evaluate the average sizes of crystalline sp2 domains and defect densities in graphene sheets) suggest that the obtained GO was characterized by a low number of defects [28]. FTIR spectroscopy and SEM imaging confirmed that both applied methods of metal coating with GO—electrochemical and chemical—can result in obtaining GO coatings that are uniform but possible to detach with time during washing. SEM images of GO coatings are similar to the results obtained by S.A. Hasan et al. [29]. The coatings obtained during these studies were also characterized by numerous wrinkles, which are promoted by the presence of oxygen-containing functional groups through the establishment of intra-sheet hydrogen bonds, and also correspond to the SEM images for GO itself, which is also characterized by numerous inequalities. The research thus confirms that the GO surface, when applied to steel, retains its visual properties in comparison to the GO itself [30]. The GO coatings made the steel surface much more hydrophilic (change of the contact angle up to approximately 50°), as the GO comprises numerous carboxyl and hydroxyl groups, and also smoothened the rough steel surface. The gold surface contact angle was not changed much. This could be caused by the different roughness of the gold surface, as gold filled the scratches on steel during material preparation through plasma sputtering. The final GO-coated materials did not exhibit much difference in contact angle regardless of the starting material and applied coating technique. There are many examples of GO-containing coatings on metals for which the contact angle has been determined in the literature. GO coatings on titanium are known, for which the contact angle was as low as 20° [31,32]. However, there are significant differences in the value of the contact angle for titanium coatings containing GO; the values vary from 20 to 70°, which may be due to the difference in the content of oxygen groups, i.e., carboxy, hydroxyl, or epoxy groups. When reduced graphene oxide was used for the coating, a contact angle of 76° was obtained [33]. Another reason may be that the metal is prepared differently before coating. For coatings containing GO on 316L steel, there are examples in the literature where the contact angle is in the range of 30° to 60° [34,35], which is a similar result to the value reported in the following study. The high contact angle for stainless steel may be related to the properties and pretreatment of the steel used. It is also worth noting that in all the cited studies, the coating containing GO on the metal caused a reduction in the contact angle.

### 4.2. Biological Properties of GO-Coated Metals

#### 4.2.1. Cell Viability

Most of the material variants were proven to be cell-compatible by showing no significant toxic effect in indirect tests apart from sample A—steel coated with GO through the electrodeposition method using GO suspended in water. It was detected with both MTT and XTT assays. This interesting remark can be explained by the differences in the durability of the obtained coatings. It has been shown in other works that GO coatings prepared on Si substrates did not stop cell proliferation as BTS cells showed linear growth during 48 h of culture [36]. Additionally, the graphene- and pHEMA-based materials showed no negative effect on HUVEC cells [30]. It has also been shown that the GO layer on steel increases the viability of MG-63 cells. The GO layer used in steel materials containing a nanofibrous PCL/Ge/forsterite layer caused an almost two-fold increase in service life compared to materials where it was not present [37]. However, many studies suggest the cytotoxicity of graphene oxide in form of suspension, which depends primarily on flake size and concentration [38]. Therefore, we suppose that the toxicity of variant A may be caused by the detachment of GO from the steel surface during incubation with a cell medium, resulting in the presence of free GO flakes in the extract (Figure 2f), which subsequently had a negative impact on the viability of L929 cells. The toxicity of graphene oxide in the solution can be caused by mechanical damage to the cell membrane by contact with the sharp edges of GO flakes [39], which is not observed in the case of GO coatings on steel. The loss in GO content visible in Figure 2f was not pointed out by FTIR spectra (Figure 2a), because it happened in the macroscale. We supposed that very thin GO coating residues were still present on the steel surface, which gave a signal for the FTIR measurement.

Other GO-coated materials did not exhibit a toxic effect on cells. The phenomenon of the detachment of graphene flakes may also be the reason for differences between the viability obtained for the other variants. However, it did not have a toxic effect on cells because it was only to a small extent. In addition, differences between the results for different variants may also result from the methodology of cytotoxicity tests, which was confirmed by tests carried out for other types of coatings, e.g., polymer coatings, where such differences were also observed [24,40,41].

#### 4.2.2. Cell Adhesion

Despite the cytotoxicity results, it was demonstrated that in direct contact, cell growth and proliferation were not negatively affected by GO coatings. In the case of steel coated with GO (A, B, and C), cells covered almost all of the available space, especially in the case of A and C samples. Whereas the extract made from sample A showed a toxic impact on cells, it was not observed in direct cell adhesion. The differences in cell morphology were most clearly seen when comparing bare steel (sample SS) to GO-coated steel (samples A, B, and C) and bare gold (Au) to GO-coated gold (D and E). Similar results were observed by A. Fraczek-Szczypta et al. In their study, GO or GO/PANI coatings were used for neurite outgrowth on titanium. It was shown that when GO is coated, it increases cell proliferation by 15% compared to uncoated titanium [32]. According to Kim et al. [42], it can be concluded that GO changed the metal surface topography. Focal adhesion points in cells growing on the surface form in the vicinity of ridges. When cells contact a surface with densely spaced ridges, they elongate rather than flatten. On the contrary, if the irregularities are distributed more sparsely, the cells take a more rounded shape. Thus, GO changed the metal surface by lowering the ridge content per surface area. Another conclusion is that more cells grew on GO-coated steel than on GO-coated gold, which was especially noticeable after 48 h of culture. The surface topography obtained with the electrodeposition of GO might have been more beneficial for L929 cells than the one achieved with covalent bonding.

#### 4.2.3. Protein Adhesion and Hemolysis

GO coatings were not albumin-repellent, despite their hydrophilicity. It is known that hydrophilic surfaces prevent nonspecific protein adsorption [40]; however, GO interacts with proteins through weak Van der Waals forces and hydrophobic, electrostatic, and π–π stacking interactions [43]. Our results indeed show that more albumin was adsorbed on GO-coated surfaces than on bare metals, so in the case of GO coatings, their hydrophilicity was not enough to prevent nonspecific protein adsorption. Importantly, the prepared materials do not break down erythrocytes. Hemolysis tests confirmed the results obtained by S. Ge et al. [17]. The coatings with an inner layer of GO loaded with docetaxel and an outer layer of carboxymethyl loaded with heparin, tested by that research group, did not cause hemolysis higher than 5%, which is following the guidelines. The research presented there also confirms that the coating on steel comprising only GO did not have a negative effect on blood erythrocytes. However, it has also been reported that suspended GO breaks down red blood cells. Liao et al. showed that suspensions of GO obtained by Hummers’ method cause hemolysis, the degree of which strongly depends on the size of the tested flakes. In the case of flakes with a size of about 350 nm, 50% lysis of erythrocytes occurred at a concentration of 20 µg/mL, while in the case of flakes with a size of about 750 nm, this concentration was 49.6 µg/mL [44]. Other studies have shown that while GO suspended in water has a hemolysis rate of about 50% when deposited on the gold surface, it drops to 2.5% [45]. In the case of the present research, it seems that small GO particles were not released from GO coatings in large quantities for any of the sample variants, nor sample A, and this is why the GO coatings were not harmful to erythrocytes. However, as previously stated, GO-coated steel (sample A) did release some amount of GO to the cell medium, which had an adverse effect on L929 viability and was the least stable during washing (Figure 2f). This was because the hemolysis test required a shorter time than extraction prior to indirect viability tests.

#### 4.2.4. The Influence of the Solvent in GO Suspension Subjected to Electrodeposition on Steel

In light of the received results, it can be concluded that variant A presented the poorest properties (detachment of GO and toxicity in indirect cell viability tests). This variant was obtained with the electrodeposition of GO suspended in water, without the addition of ethanol. These results indicate that the addition of ethanol to the GO suspension improves the quality of GO coating obtained by electrodeposition. A mixture of ethanol and water was also used for the electrodeposition of HA:GO nanopowder and chitosan on titanium. Very good results were achieved regarding the viability and adhesion of MG63 cells on these materials, which is another piece of evidence that the presence of ethanol in the electrodeposition mixture may have a positive effect on the quality of the obtained coatings [46]. It can be possible due to the fact, that in the ethanol–water mixture, there are weaker hydrogen bonds between the solvent and GO than in the case of water alone [47]. Another possible reason is better dispersibility in the ethanol–water mixture. Liu et al. ascribed the process of graphite exfoliation in the ethanol–water mixture to graphene, which was not possible in water or ethanol alone [48]. Therefore, it is suspected that in mixing ethanol with water after sonication, there are smaller particles that have a larger contact surface with steel in the electrodeposition process and form a homogeneous coating on it.

## 5. Conclusions

Both methods of coating metals with GO used in the research allowed a GO coating on the metal surface to be obtained, while the method of chemical modification on the gold surface allows for thinner layers that are almost invisible to the naked eye to be obtained (Figure 2f). The SEM images, as well as directly adhered cell morphology, show that the surfaces of coatings were smoother than bare steel and that the coatings were also more hydrophilic (contact angle around 50–70°). We examined the electrodeposition method in terms of producing high-quality GO coatings, since our previous works, cited above, have shown the potential of this technique in such applications; in the case of the present work, it seems that the choice of the solvent plays a vital role. The addition of ethanol results in the improvement of the stability of the coating. Our results indicated that GO coatings on steel and gold were not toxic towards L929 cells in a direct cell adhesion test—on all tested materials, it was possible to observe the growth of L929 cells during 48 h of culture. The lack of toxic effect on cells was also confirmed in a direct cell adhesion test and with two viability assays, XTT and MTT (viability of cells above 70%), although one of the GO coatings on steel obtained with electrodeposition (sample A) showed a slightly toxic effect, 69 ± 5.3% viability of cells. This proves that GO synthesized by our group in the form of coating is non-toxic; however, it can show toxicity if detached from the surface. The obtained materials did not show any hemolytic properties; the percentage of hemolysis was 0%, which is very promising in light of future potential applications. However, the stability of the coatings must be enhanced. This could be achieved by, e.g., covering the surface of the metal with a biocompatible inert polymer, which could be initially introduced on the metal surface with the use of electrodeposition or electropolymerization techniques. Moreover, GO coatings appeared to attract the adsorption of proteins. Because of this, GO-coated materials cannot, for instance, directly contact blood due to the high risk of thrombogenicity. Still, this does not cross out the potential for GO coatings to be applied as biocompatible surfaces, since the potential of such materials can likely be significantly expanded.

## Figures and Tables

**Table 1:** A list of symbols representing variants of material.

	Variant Description
SS	Clean stainless steel disc
A	Stainless steel disc, electrochemically coated with GO from GO suspension 0.1% *w*/*w* in water
B	Stainless steel disc, electrochemically coated with GO from GO suspension 0.1% *w*/*w* in a mixture of water and ethanol in a ratio of 1:1
C	Stainless steel disc electrochemically coated with GO from GO suspension 0.1% *w*/*w* in a mixture of water and ethanol in a ratio of 3:1
Au	Gold-surface disc
D	Gold-surface disc dip-coated with GO 0.5%
E	Gold-surface disc with chemical coating: cystamine + GO

**Table 2:** Results of hemolysis degree of obtained GO coatings in comparison to uncoated steel and gold.

Variant Number	Absorbance	±SD	%H
NC	0.040	0.0004	0
SS	0.040	0.0006	0
A	0.040	0.0005	0
B	0.039	0.0009	0
C	0.040	0.0009	0
Au	0.040	0.0014	0
D	0.040	0.0007	0
E	0.041	0.0011	0.18
PC	0.417	0.0201	100

## Data Availability

The data presented in this study are available on request from the corresponding author.

## References

[1] Hermawan H., Ramdan D., Djuansjah J.R. (2011). Metals for Biomedical Applications. Biomed. Eng. Theory Appl..

[2] Matsuno H. (2001). Biocompatibility and osteogenesis of refractory metal implants, titanium, hafnium, niobium, tantalum and rhenium. Biomaterials.

[3] Qi P., Maitz M., Huang N. (2013). Surface modification of cardiovascular materials and implants. Surf. Coat. Technol..

[4] Farooqui U., Ahmad A., Hamid N. (2018). Graphene oxide: A promising membrane material for fuel cells. Renew. Sustain. Energy Rev..

[5] Amiri A., Baghayeri M., Karimabadi F., Ghaemi F., Maleki B. (2020). Graphene oxide/polydimethylsiloxane-coated stainless steel mesh for use in solid-phase extraction cartridges and extraction of polycyclic aromatic hydrocarbons. Microchim. Acta.

[6] Manivasagam G., Dhinasekaran D., Rajamanickam A. (2010). Biomedical Implants: Corrosion and its Prevention—A Review. Recent Patents Corros. Sci..

[7] Ovcharenko E.A., Seifalian A., Rezvova M.A., Klyshnikov K., Glushkova T.V., Akenteva T.N., Antonova L.V., Velikanova E.A., Chernonosova V.S., Shevelev G.Y. (2020). A New Nanocomposite Copolymer Based on Functionalised Graphene Oxide for Development of Heart Valves. Sci. Rep..

[8] Park J.H., Park J.M. (2014). Electrophoretic deposition of graphene oxide on mild carbon steel for anti-corrosion application. Surf. Coat. Technol..

[9] Rajitha K., Mohana K.N.S., Mohanan A., Madhusudhana A.M. (2020). Evaluation of anti-corrosion performance of modified gelatin-graphene oxide nanocomposite dispersed in epoxy coating on mild steel in saline media. Colloids Surf. A Physicochem. Eng. Asp..

[10] Xu Z., Sun M., Liu Z., Wang B., Di H. (2020). Properties of the iron bacteria biofouling on Ni-P-rGO coating. Appl. Sci..

[11] Cheng W., Lu X., Kaneda M., Zhang W., Bernstein R., Ma J., Elimelech M. (2020). Graphene Oxide-Functionalized Membranes: The Importance of Nanosheet Surface Exposure for Biofouling Resistance. Environ. Sci. Technol..

[12] Fathi A.M., Ahmed M.K., Afifi M., Menazea A.A., Uskoković V. (2021). Taking Hydroxyapatite-Coated Titanium Implants Two Steps Forward: Surface Modification Using Graphene Mesolayers and a Hydroxyapatite-Reinforced Polymeric Scaffold. ACS Biomater. Sci. Eng..

[13] Kim B.-S., La W.-G., Jin M., Park S., Yoon H.-H., Jeong G.-J., Bhang S.H., Park H., Char K. (2014). Delivery of bone morphogenetic protein-2 and substance P using graphene oxide for bone regeneration. Int. J. Nanomed..

[14] Ahmed M., Menazea A., Mansour S., Al-Wafi R. (2020). Differentiation between cellulose acetate and polyvinyl alcohol nanofibrous scaffolds containing magnetite nanoparticles/graphene oxide via pulsed laser ablation technique for tissue engineering applications. J. Mater. Res. Technol..

[15] Liang C., Luo Y., Yang G., Xia D., Liu L., Zhang X., Wang H. (2018). Graphene Oxide Hybridized nHAC/PLGA Scaffolds Facilitate the Proliferation of MC3T3-E1 Cells. Nanoscale Res. Lett..

[16] Yang M.-C., Tsou H.-M., Hsiao Y.-S., Cheng Y.-W., Liu C.-C., Huang L.-Y., Peng X.-Y., Liu T.-Y., Yung M.-C., Hsu C.-C. (2019). Electrochemical polymerization of pedot–graphene oxide–heparin composite coating for anti-fouling. Polymers.

[17] Ge S., Xi Y., Du R., Ren Y., Xu Z., Tan Y., Wang Y., Yin T., Wang G. (2019). Inhibition of in-stent restenosis after graphene oxide double-layer drug coating with good biocompatibility. Regen. Biomater..

[18] Liao C., Li Y., Tjong S.C. (2018). Graphene Nanomaterials: Synthesis, Biocompatibility, and Cytotoxicity. Int. J. Mol. Sci..

[19] Loh K.P., Bao Q., Ang P.K., Yang J. (2010). The chemistry of graphene. J. Mater. Chem..

[20] Marcano D.C., Kosynkin D.V., Berlin J.M., Sinitskii A., Sun Z., Slesarev A., Alemany L.B., Lu W., Tour J.M. (2010). Improved Synthesis of Graphene Oxide. ACS Nano.

[21] Poniatowska A., Trzaskowski M., Ciach T. (2019). Production and properties of top-down and bottom-up graphene oxide. Colloids Surfaces A: Physicochem. Eng. Asp..

[22] International Organization for Standardization (2019). Biological Evaluation of Medical Devices—Part 5: Tests for In Vitro Cytotoxicity.

[23] (2012). Biological Evaluation of Medical Devices—Part 12: Sample Preparation and Reference Materials.

[24] Trzaskowska P., Poniatowska A., Tokarska K., Wiśniewski C., Ciach T., Malinowska E. (2020). Promising electrodeposited biocompatible coatings for steel obtained from polymerized microemulsions. Colloids Surf. A Physicochem. Eng. Asp..

[25] (2017). ASTM F756-17, Standard Practice for Assessment of Hemolytic Properties of Materials.

[26] Huynh N.M.N., Boeva Z.A., Smått J.-H., Pesonen M., Lindfors T. (2018). Reduced graphene oxide as a water, carbon dioxide and oxygen barrier in plasticized poly(vinyl chloride) films. RSC Adv..

[27] Hidayah N.M.S., Liu W.-W., Lai C.-W., Noriman N.Z., Khe C.-S., Hashim U., Lee H.C. (2017). Comparison on graphite, graphene oxide and reduced graphene oxide: Synthesis and characterization. AIP Conf. Proc..

[28] Liu Z., Duan X., Zhou X., Qian G., Zhou J., Yuan W. (2014). Controlling and Formation Mechanism of Oxygen-Containing Groups on Graphite Oxide. Ind. Eng. Chem. Res..

[29] Hasan S.A., Rigueur J.L., Harl R.R., Krejci A.J., Gonzalo-Juan I., Rogers B., Dickerson J.H. (2010). Transferable Graphene Oxide Films with Tunable Microstructures. ACS Nano.

[30] Pereira A.T., Henriques P.C., Schneider K.H., Pires A.L., Pereira A.M., Martins M.C., Magalhães F.D., Bergmeister H., Gonçalves I.C. (2021). Graphene-based materials: The key for the successful application of pHEMA as a blood-contacting device. Biomater. Sci..

[31] Oh J.-S., Jang J.-H., Lee E.-J. (2021). Electrophoretic Deposition of a Hybrid Graphene Oxide/Biomolecule Coating Facilitating Controllable Drug Loading and Release. Metals.

[32] Fraczek-Szczypta A., Jantas D., Ciepiela F., Grzonka J. (2020). Graphene oxide-conductive polymer nanocomposite coatings obtained by the EPD method as substrates for neurite outgrowth. Diam. Relat. Mater..

[33] Kang M.S., Jeong S.J., Lee S.H., Kim B., Hong S.W., Lee J.H., Han D.-W. (2021). Reduced graphene oxide coating enhances osteogenic differentiation of human mesenchymal stem cells on Ti surfaces. Biomater. Res..

[34] Jena G., Sofia S., Anandkumar B., Vanithakumari S., George R., Philip J. (2021). Graphene oxide/polyvinylpyrrolidone composite coating on 316L SS with superior antibacterial and anti-biofouling properties. Prog. Org. Coatings.

[35] Stango S.A.X., Vijayalakshmi U. (2021). Electrolytic deposition of composite coatings on 316L SS and its in vitro corrosion resistive behavior in simulated body fluid solution. Chem. Pap..

[36] Jeong J.-T., Choi M.-K., Sim Y., Lim J.-T., Kim G.-S., Seong M.-J., Hyung J.-H., Kim K.S., Umar A., Lee S.-K. (2016). Effect of graphene oxide ratio on the cell adhesion and growth behavior on a graphene oxide-coated silicon substrate. Sci. Rep..

[37] Khosravi F., Khorasani S.N., Khalili S., Neisiany R.E., Ghomi E.R., Ejeian F., Das O., Nasr-Esfahani M.H. (2020). Development of a Highly Proliferated Bilayer Coating on 316L Stainless Steel Implants. Polymers.

[38] Gurunathan S., Kim J.-H. (2016). Synthesis, toxicity, biocompatibility, and biomedical applications of graphene and graphene-related materials. Int. J. Nanomed..

[39] Linklater D.P., Baulin V.A., Juodkazis S., Ivanova E.P. (2018). Mechano-bactericidal mechanism of graphene nanomaterials. Interface Focus.

[40] Trzaskowska P.A., Kuźmińska A., Butruk-Raszeja B., Rybak E., Ciach T. (2018). Electropolymerized hydrophilic coating on stainless steel for biomedical applications. Colloids Surfaces B: Biointerfaces.

[41] Trzaskowska P.A., Poniatowska A., Trzaskowski M., Latocha J., Ozga P., Major R., Ciach T. (2018). Lecithin suspensions for electrophoretic deposition on stainless steel coatings. Mater. Sci. Eng. C.

[42] Kim D.-H., Han K., Gupta K., Kwon K.W., Suh K.-Y., Levchenko A. (2009). Mechanosensitivity of fibroblast cell shape and movement to anisotropic substratum topography gradients. Biomaterial.

[43] Simsikova M., Sikola T. (2017). Interaction of Graphene Oxide with Proteins and Applications of their Conjugates. J. Nanomed. Res..

[44] Liao K.-H., Lin Y.-S., Macosko C.W., Haynes C. (2011). Cytotoxicity of Graphene Oxide and Graphene in Human Erythrocytes and Skin Fibroblasts. ACS Appl. Mater. Interfaces.

[45] Cai B., Hu K., Li C., Jin J., Hu Y. (2015). Bovine serum albumin bioconjugated graphene oxide: Red blood cell adhesion and hemolysis studied by QCM-D. Appl. Surf. Sci..

[46] Karimi N., Kharaziha M., Raeissi K. (2019). Electrophoretic deposition of chitosan reinforced graphene oxide-hydroxyapatite on the anodized titanium to improve biological and electrochemical characteristics. Mater. Sci. Eng. C.

[47] Neklyudov V.V., Khafizov N.R., Sedov I., Dimiev A.M. (2017). New insights into the solubility of graphene oxide in water and alcohols. Phys. Chem. Chem. Phys..

[48] Liu W.-W., Xia B.-Y., Wang X.-X., Wang J.-N. (2012). Exfoliation and dispersion of graphene in ethanol-water mixtures. Front. Mater. Sci..

